# Position-Specific External Load Between Official Matches and Small-, Medium- and Large-Sided Games in Professional Football

**DOI:** 10.3390/jfmk11030306

**Published:** 2026-08-03

**Authors:** Lukas Herzig, Jörg Stöckert, Matthias Lochmann, Michael C. Rumpf

**Affiliations:** 1 Department of Sport Science and Sport, Friedrich Alexander University Erlangen-Nürnberg, 91058 Erlangen, Germany; lukas.herzig@fau.de (L.H.);; 2 1. FC Nürnberg e.V., 90480 Nürnberg, Germany; 3 Sport Performance Research Institute New Zealand, AUT-University, Auckland 1142, New Zealand; 4 Footballscience.net, 63303 Dreieich, Germany

**Keywords:** soccer, small-sided games, conditioning, load management

## Abstract

**Objectives:** Sided games are commonly used to condition players for the demands of official games (OGs). Therefore, this study aimed to evaluate if different sided games match position-specific locomotor and mechanical OG demands of professional football players. **Methods**: Twenty-one male professional football players from a second team of a professional football club were monitored during the second half of the 2024/25 season. External load was assessed during OGs and sided games. The latter were categorized by area per player (ApP) as small- (SSG, ApP < 100 m^2^), medium- (MSG, ApP 100–200 m^2^) and large-sided games (LSG, ApP > 200 m^2^). External load was analyzed for total distance (TD), high-speed running (HSR), sprint distance (SPD), and the number of accelerations (ACC) and decelerations (DEC). Players were grouped by position: center backs (CBs), full backs (FBs), central midfielders (CMs), wide midfielders (WMs), and strikers (STs). **Results**: Across all playing positions (CB, FB, CM, WM, ST), SSGs, MSGs and LSGs elicited significantly higher TD than the OG, except for CM, where no significant differences were observed during SSGs and LSGs. All positions displayed similar HSR during LSGs than OG demands, and some positions also during MSGs (CB, CM, FB) and SSGs (CB). SPD was similar across all positions only during LSGs. ACC and DEC were significantly higher for all positions in SSGs and MSGs than OGs and similar in LSGs. **Conclusions**: Position-specific OG demands were not statistically from different sided-game formats. ACC, DEC and TD in LSGs met OG demands; however, smaller formats (SSGs, MSGs) overload those variables. In contrast, HSR and SPR load in SSGs and MSGs did not meet OG demands for most positions. For that, coaches should use LSGs, knowing that TD may be overloaded, or supplement SSGs/MSGs with running-based drills for more accurate programming.

## 1. Introduction

Athletic performance is a key component of football players [1,2]. Therefore, a central purpose of football training sessions is to prepare players for the demands of official games (OGs) [1,2]. Monitoring players’ external load during OGs and training sessions with the use of Global Positioning Systems (GPS) is a well-established method [3,4] and enables practitioners to prescribe training load with greater objectivity [5]. Adequate volume of mechanical and locomotor load, especially high-speed-running (HSR) and sprinting distance (SPD), was shown to enhance players’ physical performance levels and prevent injuries [6,7,8,9]. Consequently, OG demands are suggested to serve as baseline for training load prescription [3,10] and significant positional differences were found for locomotor and mechanical variables during OGs [11,12,13,14]. This highlights the need for training load individualization, and, depending on the daily training goal [5], locomotor and mechanical loads should be targeted using percentage of OGs for each metric [2,4].

Whilst targeting physical conditioning, sided games are implemented within the training microcycle, as they also require technical and tactical capabilities [15]. A key modulator of physical output in such games is the area per player (ApP) [15,16]. A recent review by Rumpf et al. [17] stated that an increase in ApP results in higher physical output for locomotor variables (e.g., total distance (TD), HSR, and SPD), while mechanical variables such as the number of accelerations (ACC) and decelerations (DEC) remain relatively stable regardless of pitch size [17,18]. In this context, Riboli et al. [10] calculated a specific ApP to replicate position-specific OG demands for TD, HSR and SPD in professional football players. The findings demonstrated positional differences for TD with ApP ranging from 165 and 214 m^2^, while even larger positional differences were found for HSR (>15 km/h) and SPD (>24 km/h). Specifically, the ApP varied substantially for HSR and SPD for center backs (CBs; 205 m^2^, 278 m^2^), full backs (FBs; 246 m^2^, 274 m^2^), central midfielders (CMs; 311 m^2^, 340 m^2^), wide midfielders (WMs; 222 m^2^, 281 m^2^), and forwards (FWs; 333 m^2^, 407 m^2^). Additionally the ApP to replicate OG demands varies across both age category [19] and competitive level [20]. Riboli et al. [19] suggested that in professional youth football (U15–U19) an ApP between 200 and 300 m^2^ is required to replicate HSR and SPD, whereas a smaller ApP may be sufficient for TD (158 m^2^) and ACC/DEC (156 m^2^). Importantly, the specific ApP required was influenced by both age category and playing positions. Specifically, U15/16 players require a larger ApP than older age groups [19]. Positional differences in the ApP were observed for all locomotor variables; however, no consistent position-specific trends were found across different age categories. Therefore, the authors suggested that the ApP should be individualized according to age group and position [19].

As it seems, the ApP receives strategic importance during soccer training regimes, and consequently sided games can be categorized into small- (SSG), medium- (MSG) and large- (LSG) sided games, defined by under 100 m^2^, 100–200 m^2^ and over 200 m^2^, respectively [21,22]. As per this classification, LSGs would be necessary to replicate OG locomotor demands for all positions [10]. However, Sydney et al. [23] found that SSGs with an ApP of 68–125 m^2^ and MSGs with an ApP of 154–183 m^2^ replicated or even exceeded TD demands across all positions utilizing smaller formats of 3 vs. 3 and 5 vs. 5 (in SSGs) and 6 vs. 6 and 7 vs. 7 (in MSGs) respectively. Based on these circumstances, smaller sided-game formats (SSGs/MSGs) may be sufficient to replicate OG demands. In contrast, HSR (>19.8 km/h) was significantly lower than OGs during LSGs for FBs, CMs and STs, whereas WM achieved comparable HSR [23]. However, MSGs were sufficient to replicate HSR demands for CBs [23]. This indicates a mismatch of positional external load between different sided-game formats and OG for most of the aforementioned positions. Noteworthy, this study was conducted with U17 players, limiting transferability to adults [23]. Despite the widespread use of SSGs, MSGs and LSGs in football training, the position-specific locomotor and mechanical demands of OGs across these game formats has not been investigated in professional football players competing over an entire half-season. Accordingly, this exploratory study aimed to examine the extent to which SSGs, MSGs and LSGs replicate the position-specific locomotor and mechanical demands of OGs in professional football players in a reserve team.

## 2. Materials and Methods

### 2.1. Participants

Twenty-one male professional football players (21.4 ± 3.00 years, 183 ± 4.20 cm, 78.5 ± 6.70 kg) of the reserve squad of a professional German football club participated in this investigation. The parent club’s first team plays in the 2nd highest division (2. Bundesliga), while the reserve team competed in the 4th national division, played one OG and trained five times per week. The average weekly training duration was 5:15 ± 0:19 h.

All players adhered to the team’s standard training and competition schedule. Inclusion criteria required players to be officially registered with the club and injury free and the completion of at least 80% of the training sessions during the observation period. Exclusion criteria included: (1) incomplete participation during study period; (2) injury or illness limiting full participation; and (3) playing position as goalkeeper (GK), due to the distinct physical and tactical demands of this role. Only players who started and completed the entire match were included for OG analysis. Players were categorized by the head coach according to the individual playing position: CB, (N = 4), FB (N = 4), CM (N = 6), WM (N = 3), ST (N = 4).

The data used in this research originated from routine daily training monitoring practices and were obtained retrospectively from the club’s performance database. Prior to analysis, all player data were de-identified to ensure confidentiality and to prevent the identification of individual players or the club [24,25]. Therefore, formal ethics approval was not deemed necessary [26]. Nevertheless, the study was conducted in strict accordance with the ethical standards outlined in the Declaration of Helsinki, safeguarding the confidentiality of all players and the club [24]. Additionally, data processing and handling complied with the applicable data protection regulations. The club confirmed that data collection, storage, and use for scientific purposes complied with the applicable local legislation and data protection requirements.

### 2.2. Procedures

The study was conducted during the second half of the competitive 2024/2025 season from February to May. In total, 16 OGs, five different SSGs, five different MSGs and nine different LSGs were included for data analysis. Training sessions followed the team’s microcycle periodization, with SSGs and MSGs performed on match day minus four (MD-4) and LSGs on match day minus three (MD-3), which is a common periodization strategy in football [6]. All sessions were held on a natural grass pitch at the same time of the day to minimize circadian and seasonal variability. Detailed information on the sided-game formats, their number of bouts, and bout and recovery durations is displayed in Table 1.

To promote continuous play during sided games, balls were always made available directly after interruption [21]. To maintain high work rates, verbal encouragement was used [23]. Players were instructed to play with their assigned positional roles during all sided-game formats. Across all formats, a total of 1099 individual observations were recorded. Specifically, the number of individual observations was 109 for OGs (CB: 25, FB: 18, CM: 30, WM: 11, ST: 25), 350 for SSGs (CB: 72, FB: 54, CM: 111, WM: 48, ST: 65), 289 for MSGs (CB: 61, FB: 48, CM: 76, WM: 30, ST: 74) and 351 for LSGs (CB: 79, FB: 62, CM: 100, WM: 49, ST: 61).

### 2.3. Data Collection

External load data were collected using 10 Hz GPS (Catapult Vector S7, Melbourne, Australia) units. Each unit was activated at least 15 min before each training session or OG, to allow for sufficient satellite acquisition, as per manufacturers’ guidelines. To minimize inter-unit differences, each player used the same GPS unit consistently throughout the investigation as previously recommended by Riboli et al. [10]. GPS devices were positioned between the shoulder blades using the manufacturers’ supplied vests. After each training session and OG, raw GPS data were exported and processed using the corresponding software (OpenFieldConsole, Version 3.9, Catapult, Melbourne, Australia). The system has demonstrated strong validity and reliability for monitoring locomotor and mechanical output [27,28,29]. Excellent inter- and intra-system reliability was documented with an intraclass correlation coefficient (ICC) of 0.91 for ACC and 0.97 for DEC [28]. Similarly, starting velocity (0.96), maximal sprinting speed (0.97) and peak power output (0.94) were also shown to have a very large to nearly perfect reliability [27]. Thresholds and categories for mechanical and locomotor variables were adopted from the previous literature [30]: ACC > 2.5 m/s^2^ and DEC < −2.5 m/s^2^. TD was the total distance covered. HSR and SPD were the distance covered between 19.8 to <25.2 km/h and ≥25.2 km/h, respectively. Relative values per minute were calculated for each variable.

### 2.4. Statistical Analysis

All statistical analyses were performed using R (Version 4.5.0, R Core Team, Posit PBC, Boston MA, USA) with RStudio (Version 2025.02.0+496; R Core Team, Posit PBC, Boston MA, USA). Descriptives are presented as mean ± SD. Differences between game formats (SSG, MSG, LSG) and OGs were statistically investigated utilizing linear mixed models (LMMs) using the lme4 package [31]. LMM is well suited for repeated measure designs [31,32]. Normality was verified using visual inspection of Q-Q plots, while homoscedasticity was checked through residuals vs. fitted values plots. Playing format, playing position, and their interaction were included as fixed effects, while player identity was specified as a random intercept to account for repeated measurements [21]. Interaction effects were examined first. When no significant interaction was observed, the interaction term was removed and the main effects were interpreted. Post hoc pairwise comparisons were adjusted using the Bonferroni correction. Cohen’s d effect size (ES) with 95% confident interval (CI) was calculated and interpreted as trivial (*d* < 0.10), small (*d* = 0.10–0.29), medium (*d* = 0.30–0.49), large (*d* = 0.50–0.69) or very large (*d* > 0.70) [33]. Significance level was set at *p* < 0.05.

## 3. Results

Values for all variables of OG and sided-game formats across each playing position are displayed in Table 2. The full model including the format × position interaction was tested, but showed no statistical significance.

TD was significantly higher (*p* < 0.001) in SSGs, MSGs and LSGs than OGs for all positions, with very large effects (*d* = 0.74–1.84) observed. The only exception was CM, for whom significantly higher values were only displayed during MSGs (*p* < 0.001, d = 0.87, very large ES), while TD did not differ significantly between OGs and LSGs or SSGs. HSR was significantly lower in SSGs than OGs (*p* < 0.05) across all positions, except CB, with ES ranging from large to very large (*d* = −0.56–−1.73). In contrast to the other positions, ST and WM exhibited significantly lower HSR values (*p* < 0.05) during MSGs compared to OGs, corresponding to very large effects (*d* = −0.80–−0.92). HSR did not differ significantly between LSGs and OGs across all positions. Similarly, SPD did not differ significantly between OGs and LSGs across all positions, whereas during SSGs and MSGs significantly lower values were observed (*p* < 0.05), with very large effects throughout (*d* = −0.80–−3.15). ACC and DEC were significantly higher during SSGs and MSGs compared to OGs across all positions (*p* < 0.001) with very large ES (*d* = 1.19–2.30), while no significant differences were found for LSGs across all positions.

## 4. Discussion

The aim of this study was to evaluate if different sided games match position-specific locomotor and mechanical OG demands of professional football players. SSGs and MSGs displayed significantly higher ACC, DEC, and TD (except CM during SSG) than OGs across all positions, while SPD was significantly lower in those formats. HSR was significantly lower during SSGs (except for CB) and MSGs (except CB, CM and FB) compared to OGs.

TD was significantly higher across SSGs, MSGs and LSGs and positions compared to OGs except for CM. For the latter, only MSGs displayed significantly higher values compared to OGs. Riboli et al. [10] suggested an ApP of 187 m^2^ (165–214 m^2^) to meet OG demands for TD. In contrast, the present study found that, except for CM (in SSGs and LSGs), all sided-game formats displayed significantly higher TD OG demands. This is similar to the findings by Sydney et al. [23], where all SSGs, MSGs and LSGs (ApP ranging from 68 m^2^ in a 3 vs. 3 format to 243 m^2^ in a 9 vs. 9 format) displayed significant higher TD values compared to OGs across all positions in youth football players. Moreover, in a study by Martín-Garcia et al. [34], SSGs with an ApP of 88 m^2^ (6 vs. 6 + 1) and 110 m^2^ (5 vs. 5) produced similar TD values to OGs across all positions in professional football players. This suggests that such sided-game formats may be used to reproduce similar or even higher values than OGs across all positions. It is important to note that TD tends to decrease with a smaller ApP [16,17,18] and the smallest ApP used across studies was 68 m^2^ [23]. Consequently, a further reduction in ApP may no longer reproduce TD OG demands.

HSR exhibited the greatest positional variation in sided games compared to OGs. CB achieved comparable HSR values already in SSGs (ApP < 100 m^2^), CM and FB in MSGs (ApP 100–200 m^2^), while ST and WM required LSGs (ApP > 200 m^2^) to meet OG demands. LSGs proved to be the most effective format in replicating OG HSR across all positions in the current investigation, supporting the findings of Riboli et al. [10] who stated that an ApP between 205 m^2^ and 333 m^2^ is necessary in professional football players. In contrast, Sydney et al. [23] found that ST experienced significantly lower HSR values during LSGs compared to OGs in youth football. However, their ApP used during LSGs was smaller (240 m^2^ and 270 m^2^) than recommended by Riboli et al. [10] and the one in this study (ApP 268–324 m^2^). This emphasizes the necessity of an ApP larger than 270 m^2^ to receive similar HSR OG demands for ST. Interestingly, we found that positions with the lowest HSR OG values (CB, CM) reproduced HSR already during SSGs (CB) or MSGs (CM). The present results align with previous scientific investigations that CB require a smaller ApP compared to all other positions to match HSR demands [10,23]. However, the specific format to receive similar HSR differed between the present study (SSG > 100 m^2^) and the study by Sydney et al. [23], where MSGs (ApP: 154–182 m^2^) were required in youth football players. Additionally, Riboli et al. [10] suggested an ApP of 205 m^2^, which would formally be classified as an LSG. It is important to note that this is only marginally larger (+5 m^2^) than the threshold for MSGs (100–200 m^2^). The reduced ApP required by CBs may reflect their distinct tactical role during OGs, where they typically maintain a central and defensive role within in the pitch, limiting their involvement in offensive and transitional phases, resulting in lower HSR exposure [14]. This positional effect may be attenuated during SSGs and MSGs, where positional roles are less constrained, resulting that HSR is less influenced by position in SSGs and MSGs [35], leading to a higher relative load for CBs.

CMs and FBs experienced similar OG HSR demands during MSGs in this investigation. However, both positions were found to replicate HSR demands only in LSGs (ApP: 311 m^2^, 246 m^2^) in the study by Riboli et al. [10], while LSGs even displayed significantly lower values (small effect) in youth footballers in the study by Sydney et al. [23]. A possible explanation of these discrepancies might be the lower number of players used during MSGs in this study. Specifically, only 4 vs. 4 and 5 vs. 5 formats were employed in this study, whereas Riboli et al. [10] utilized 5 vs. 5, 6 vs. 6, 7 vs. 7, 9 vs. 9 and 10 vs. 10 for their regression analysis and Sydney et al. [23] used 6 vs. 6 and 7 vs. 7 formats during their MSGs in youth players. It is argued that lower player number reduces tactical organization and positioning plays and players are more likely to enter unusual spaces, resulting in higher HSR exposure [23]. However, this also represents a confound, as it becomes difficult to disentangle whether the observed differences in HSR replication are driven by the number of players itself or by the format including ApP. Still, this might explain why MSGs were sufficient to match HSR demands not only for CBs but also for CMs and FBs in this study. However, LSGs are commonly employed by practitioners to condition players for HSR demands [5], as it is believed that these games maintain a high technical [36] and tactical [37,38] specificity to OGs. The results of this study support this practice, as not all players experienced OG demands during smaller formats (SSGs, MSGs). Consequently, if such formats (SSGs, MSGs) are used and HSR exposure is insufficient (e.g., for ST, WM) additional training methods (e.g., running-based drills) are required to ensure adequate HSR load.

SPD was found to be significantly lower in SSGs and MSGs compared to OG across all positions. In line with previous research, LSGs are required for all players to reach SPD demands [10,36,39]. Accordingly, LSGs provide an effective format for replicating SPD across all positions. Nevertheless, CMs and STs exhibited non-significantly lower values in LSGs compared to OGs, while other positions (CB, FB, WM) displayed non-significantly higher values than OG demands. A similar trend was previously observed by Riboli et al. [10] who found that a minimum ApP of 278, 274, and 281 m^2^ was needed to replicate SPD for CBs, FBs, and WMs respectively. CM and ST required 340 m^2^ and 407 m^2^ which is higher than the ApP used in the present study (≈268–320 m^2^).

However, given the importance of adequate SPD exposure for injury prevention [7], it is essential to monitor SPD closely during training sessions and adjust ApP accordingly or supplement with additional training [6,40]. It was recently suggested by Gomez-Piqueras and Alcaraz [6] that coaches should prescribe the weekly sprint dose according to playing position and OG demands. For example, in this study STs were recommended to accumulate 11–16 sprint efforts, corresponding to 200–260 m of SPD per week [6]. When these targets are not achieved during regular training, coaches are advised to implement supplementary running-based drills. Examples include maximal-effort 45 m (15 m acceleration + 30 m sprint) flying sprint repetition [6] or sets of 50 m shuttle runs completed in 7–8 s, with 21–24 s of passive recovery [41]. The prescription of these drills should be individualized according to the position-specific OG requirements to avoid excessive overload [42].

ACC and DEC demands were met across all formats and positions, though SSGs and MSGs displayed significantly higher values compared to OGs over all positions. This is consistent with previous findings that SSGs [21,34,43] and MSGs [34,41,43] elicit significant higher ACC and DEC than OGs for all positions. Furthermore, both SSGs and MSGs were found to produce greater ACC and DEC values compared to LSGs [34,35] for all positions.

It must be noted that ACC and DEC profiles generally differ across playing positions. FBs have been reported to perform a greater number of low-intensity ACC (0.5–2.0 m/s^2^), while CMs reported the greatest number of high intensity DEC (−2–−10 m/s^2^) [44]. Despite these positional differences, such magnitudes of ACC have been shown to be replicated within the microcycle; however, DEC was less likely to be reproduced [44]. Most studies investigating ACC demands have applied thresholds between 2.0 and 3.0 m/s^2^ potentially overlooking this positional difference. For ACC, an inverse relationship between starting velocity and ACC magnitude has generally been reported across different thresholds [22], indicating that higher ACC counts are achieved from initial lower velocities in smaller-sided formats (e.g., SSG/MSG) [16,19]. In contrast, DEC behaviors appear different. While smaller pitch sizes elicit in a greater number of DEC of “lower” intensities (<−4 m/s^2^), higher-intensity DEC (<−4 m/s^2^) has been found to occur more frequently when the pitch size is enlarged [16]. This may be explained by the requirement for players to attain higher running velocities before executing substantial braking actions, thereby increasing the likelihood of achieving greater DEC magnitudes [22].

Overall, these findings suggest that LSGss are suitable to meet OG demands of ACC/DEC, while SSGs and MSGs may elicit even greater ACC and DEC values across all positions. However, caution is warranted when considering high intensity DEC (e.g., <−4 m/s^2^), which may be less reproducible during smaller formats. Therefore, practitioners may select a format that aligns with their technical or tactical objectives [17]; however, they need to be cautious and control excessive load in SSGs and MSGs. For example, an increase in ApP leads to less ball touches, dribbles, turnover and shots [45], but higher ball possession [17,18,46], stretch index [17,18] and interpersonal distance [17]. This study is not without limitations. First, no physiological measures were investigated in this study. Additionally, the use of different thresholds for locomotor and mechanical variables throughout the scientific literature may limit the direct comparability of results. However, mechanical outcomes remained consistent irrespective of threshold utilization. Moreover, the study was restricted to a single professional football team and a relatively low number of positional subgroups, which may limit the generalizability of the findings. Finally, as SSGs and MSGs were performed on MD-4 and LSGs on MD-3, residual fatigue from the preceding training sessions may have influenced the external load achieved during LSGs [47]. However, despite this possibility, HSR, SPD, ACC, and DEC did not differ significantly between MD-3 and OGs across any playing position, while total distance (TD) was significantly higher during MD-3. Nevertheless, OGs are typically preceded by a tapering phase that allows for more complete recovery [47]. Therefore, comparisons between sided-game formats and OG demands should be interpreted within the context of this specific microcycle and training regimen and cannot be compared to other games such as positional games due to methodological differences.

## 5. Conclusions

The findings provide valuable insights for coaches seeking to meet OG demands utilizing a variety of sided games. Coaches can use the smaller formats (SSGs, MSGs) to match OG demands regardless of position for ACC, DEC and TD. However, these variables tend to overload players compared to OGs and therefore should be monitored closely. HSR showed positional differences and different pitch sizes are recommended to reproduce OGs for specific positions. While this seems cumbersome for team training, coaches may use LSGs to ensure HSR demands are met for all positions. Concomitant OG SPDs were met in LSGs. However, supplementary drills (e.g., running-based drills) can also be implemented, if necessary or desired. However, these findings should be interpreted in light of contextual factors, such as the inclusion of a single professional football team from one competitive level and the relatively small positional sample size. To enhance the external validity of the findings, future research should replicate this design across multiple teams, populations (e.g., female football players), competitive levels (e.g., different leagues) and age groups (e.g., youth team), while incorporating larger sample sizes. Finally, integrating physical and physiological (e.g., heart rate, biomarkers) measures would provide a more comprehensive understanding of performance demands.

## 6. Practical Application

**Figure 1:**
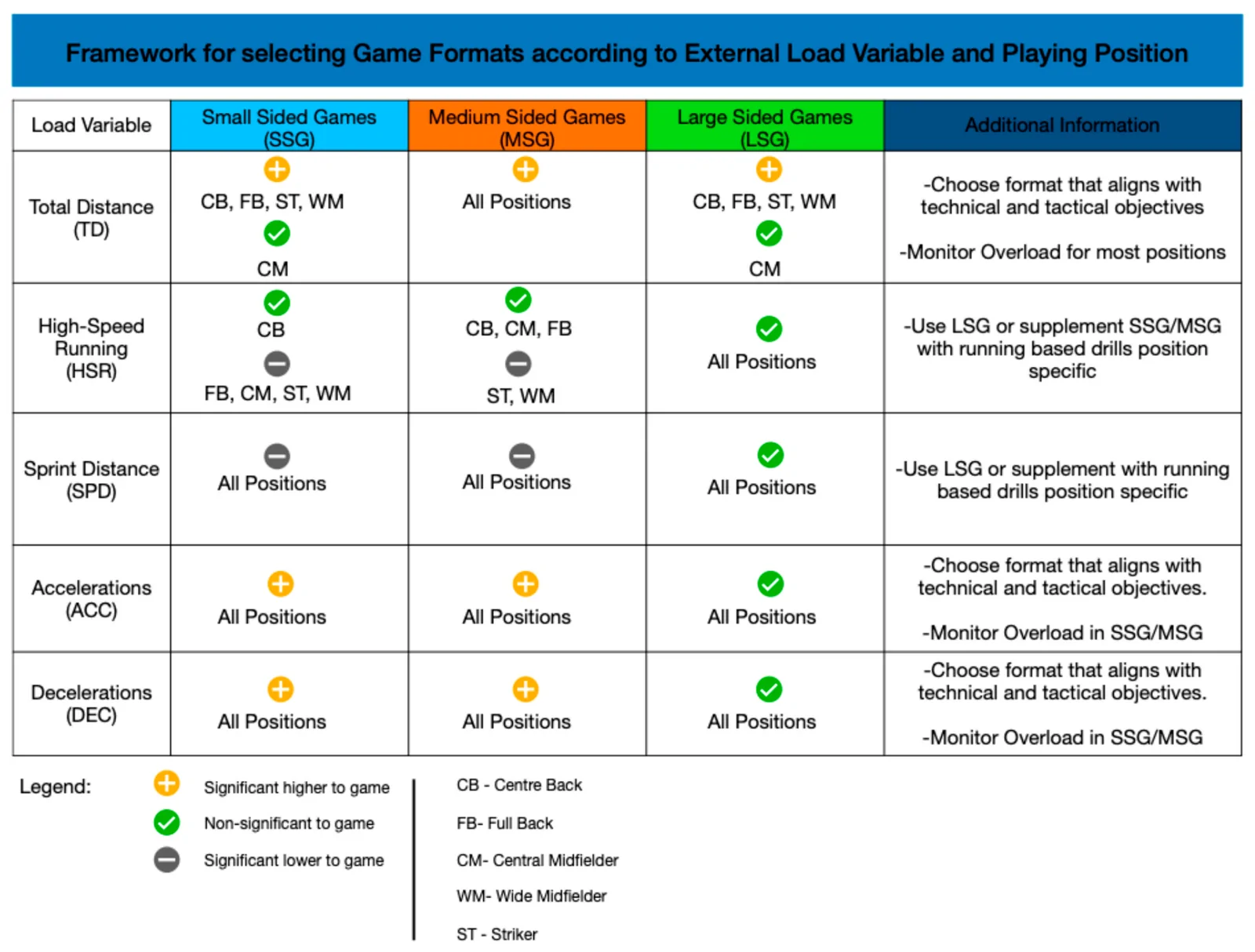
Framework for selecting game formats according to external load variable and playing position. The load variables are listed in the first column and a specific variable should be selected according to the desired training purpose. Once the variable is selected, the different game format columns (small-, medium-, large-sided game) indicate position-specific significantly lower (gray minus), non-significant equal (green checkmark), or significantly higher (orange plus) loads within the same row.

While Table 2 remains the primary data source, Figure 1 summarizes several practical applications deriving from this study:

- TD is consistently overloaded across sided-game formats across most playing positions and should therefore be monitored.
- ACC/DEC are overloaded during SSGs/MSGs and are similar in LSGs.
- Coaches should monitor ACC/DEC during SSGs/MSGs, as an overload of such action contributes to muscle damage and fatigue.
- Use LSGs to stimulate HSR and SPD for all positions or supplement SSGs/MSGs with running-based drills that are position-specific.

## Figures and Tables

**Table 1:** Various sided-game format, pitch size, area per player, the number of bouts, bout duration and rest between bouts.

	Format	Pitch Size (m)	Area per Player (m^2^)	Number of Bouts	Bout Duration (min)	Between Set Rest (min)
SSG	4 vs. 4	36 × 22	79	6	3–4 min	2–3 min
	4 vs. 4	36 × 26	88	3	2–3 min	2 min
	4 vs. 4	40 × 23	92	6	3–4 min	2–3 min
	4 vs. 4	33 × 28	92	6	2–3 min	2–3 min
	4 vs. 4	34 × 29	98	3	3–4 min	2–3 min
MSG	4 vs. 4	30 × 35	105	4	3–4 min	2–4 min
	4 vs. 4	30 × 49	147	3	3–4 min	2–3 min
	5 vs. 5	36 × 49	147	6	2–3 min	1–2 min
	5 vs. 5	40 × 50	166	4	3–4 min	2–3 min
	5 vs. 5	47 × 49	191	3	4–5 min	2–3 min
LSG	7 vs. 7	67 × 67	280	3	10–12 min	3–5 min
	8 vs. 8	72 × 67	268	2	10–13 min	5 min
	9 vs. 9	94 × 67	314	2	15–16 min	4 min
	9 vs. 9	95 × 67	318	3	10–13 min	3–5 min
	9 vs. 9	95 × 67	318	3	10–15 min	3–5 min
	10 vs. 10	105 × 67	320	2	16–22 min	5 min
	10 vs. 10	105 × 67	320	4	11–13 min	3–5 min
	10 vs. 10	105 × 68	324	1	23 min	-
	10 vs. 10	105 × 67	320	2	20–21 min	5 min

**Table 2:** Total distance (TD, m/min), high-speed running (HSR, m/min), sprint distance (SPD, m/min), accelerations (ACC, n/min) and decelerations (DEC, n/min), during small- (SSG), medium- (MSG) and large- (LSG) sided games and game by playing position.

Format	Position	TD	*d* (95% CI)	HSR	*d* (95% CI)	SPD	*d* (95% CI)	ACC	*d* (95% CI)	DEC	*d* (95% CI)
SSG	FB	124 ± 12.7 **	1.06 (0.49–1.62)	3.96 ± 3.37 *	−0.93 (−1.50–0.37)	0.05 ± 0.35 **	−3.15 (−3.91–−2.40)	1.92 ± 0.63 **	2.30 (1.64–2.97)	1.68 ± 0.74 **	1.59 (0.90–2.10)
MSG		130 ± 20.0 **	1.07 (0.49–1.64)	6.10 ± 5.52	−0.14 (−0.69–0.41)	0.25 ± 0.76 **	−1.57 (−2.18–−0.95)	1.69 ± 0.80 **	1.53 (0.92–2.14)	1.61 ± 0.77 **	1.34 (0.74–1.94)
LSG		118 ± 9.53 **	0.74 (0.20–1.28)	7.79 ± 3.01	0.37 (−0.17–0.90)	1.72 ± 1.40	0.28 (−0.26–0.81)	0.81 ± 0.26	0.79 (0.24–1.33)	0.89 ± 0.31	0.66 (0.11–1.20)
Game		111 ± 6.54		6.78 ± 1.48		1.37 ± 0.58		0.61 ± 0.22		0.70 ± 0.23	
SSG	CM	119 ± 10.9	0.63 (0.22–1.04)	2.74 ± 2.60 *	−0.82 (−1.24–0.40)	0.08 ± 0.59 **	−1.28 (−1.72–−0.85)	1.67 ± 0.73 **	1.83 (1.37–2.29)	1.54 ± 0.75 **	1.37 (0.93–1.81)
MSG		123 ± 14.1 **	0.87 (0.43–1.31)	3.65 ± 4.04	−0.31 (−0.74–0.12)	0.06 ± 0.43 **	−1.59 (−2.07–−1.11)	1.60 ± 0.85 **	1.56 (1.08–2.03)	1.43 ± 0.72 **	1.31 (0.84–1.77)
LSG		119 ± 16.3	0.66 (0.24–1.08)	4.64 ± 2.50	−0.03 (−0.45–0.38)	0.51 ± 0.71	−0.46 (−0.88–0.05)	0.70 ± 0.31	0.84 (0.41–1.26)	0.72 ± 0.29	0.41 (0.00–0.83)
Game		112 ± 9.17		4.76 ± 1.81		0.84 ± 0.61		0.47 ± 0.16		0.62 ± 0.18	
SSG	CB	125 ± 15.2 **	1.48 (0.97–1.98)	3.47 ± 4.03	−0.09 (−0.56–0.37)	0.14 ± 0.63 **	−0.90 (−1.38–−0.42)	1.67 ± 0.63 **	2.08 (1.53–2.63)	1.47 ± 0.72 **	1.56 (1.04–2.07)
MSG		125 ± 21.3 **	1.50 (0.98–2.02)	3.50 ± 3.99	−0.07 (−0.54–0.40)	0.15 ± 0.70 *	−0.80 (−1.29–−0.31)	1.59 ± 1.01 **	1.29 (0.78–1.81)	1.34 ± 0.86 **	1.21 (0.70–1.72)
LSG		118 ± 9.75 **	1.46 (0.96–1.95)	4.67 ± 2.13	0.45 (−0.01–0.91)	0.79 ± 0.95	0.16 (−0.30–0.61)	0.68 ± 0.27	0.60 (0.14–1.07)	0.72 ± 0.32	0.81 (0.35–1.28)
Game		105 ± 6.24		3.80 ± 1.17		0.65 ± 0.35		0.54 ± 0.20		0.50 ± 0.16	
SSG	WM	126 ± 12.3 **	1.42 (0.71–2.12)	4.99 ± 4.27 *	−0.56 (−1.24–0.12)	0.23 ± 0.73 **	−2.80 (−3.65–−1.96)	2.05 ± 0.88 **	1.69 (0.95–2.43)	2.15 ± 0.75 **	1.85 (1.10–2.60)
MSG		133 ± 14.3 **	1.84 (1.03–2.64)	4.51 ± 3.80 *	−0.80 (−1.54–0.07)	0.52 ± 1.46 **	−1.40 (−2.18–−0.62)	2.06 ± 0.91 **	1.73 (0.92–2.54)	2.11 ± 0.65 **	2.18 (1.32–3.05)
LSG		118 ± 10.7 **	0.88 (0.20–1.56)	7.88 ± 2.60	0.30 (−0.37–0.97)	2.84 ± 1.96	0.24 (−0.43–0.91)	0.98 ± 0.40	0.78 (0.10–1.47)	1.20 ± 0.36	0.96 (0.27–1.65)
Game		109 ± 6.50		7.16 ± 0.68		2.40 ± 0.97		0.69 ± 0.24		0.88 ± 0.13	
SSG	ST	120 ± 11.6 **	1.16 (0.67–1.66)	2.23 ± 2.97 **	−1.73 (−2.26–−1.20)	0.09 ± 0.46 **	−2.40 (−2.99–−1.81)	1.48 ± 0.80 **	1.19 (0.69–1.69)	1.61 ± 0.76 **	1.26 (0.75–1.76)
MSG		128 ± 16.0 **	1.44 (0.95–1.94)	3.93 ± 3.49 **	−0.92 (−1.40–−0.44)	0.20 ± 0.64 **	−2.00 (−2.54–−1.46)	1.75 ± 0.87 **	1.45 (0.94–1.95)	1.77 ± 0.85 **	1.33 (0.83–1.82)
LSG		119 ± 10.3 **	1.21 (0.71–1.72)	6.26 ± 2.06	−0.27 (−0.75–0.20)	1.12 ± 1.31	−0.51 (−0.99–0.03)	0.65 ± 0.29	0.02 (−0.49–0.46)	0.92 ± 0.30	0.49 (0.01–0.97)
Game		108 ± 6.93		6.77 ± 1.34		1.76 ± 1.09		0.65 ± 0.18		0.77 ± 0.16	

*p* < 0.05 (*), *p* < 0.001 (**).

## Data Availability

The data presented in this study are available on reasonable request from the corresponding author.

## References

[1] Douchet T., Michel A., Verdier J., Babault N., Gosset M., Delaval B. (2025). Intensity vs. Volume in Professional Soccer: Comparing Congested and Non-Congested Periods in Competitive and Training Contexts Using Worst-Case Scenarios. Sports.

[2] Ammann L., Altmann S. (2023). Training and Match Load Ratios in Professional Soccer–Should We Use Player- or Position-Specific Match Reference Values?. Front. Sports Act. Living.

[3] Riboli A., Nardi F., Osti M., Cefis M., Tesoro G., Mazzoni S. (2025). Training Load, Official Match Locomotor Demand, and Their Association in Top-Class Soccer Players During a Full Competitive Season. J. Strength. Cond. Res..

[4] Ravé G., Granacher U., Boullosa D., Hackney A.C., Zouhal H. (2020). How to Use Global Positioning Systems (GPS) Data to Monitor Training Load in the “Real World” of Elite Soccer. Front. Physiol..

[5] Buchheit M., Douchet T., Settembre M., Mchugh D., Hader K., Verheijen R. (2024). Microcycle Periodization in Elite Football The 11 Evidence-Informed and Inferred Principles of Microcycle Periodization in Elite Football. Sport Perf. Sci. R..

[6] Gómez-Piqueras P., Alcaraz P.E. (2024). If You Want to Prevent Hamstring Injuries in Soccer, Run Fast: A Narrative Review about Practical Considerations of Sprint Training. Sports.

[7] Malone S., Owen A., Mendes B., Hughes B., Collins K., Gabbett T.J. (2018). High-Speed Running and Sprinting as an Injury Risk Factor in Soccer: Can Well-Developed Physical Qualities Reduce the Risk?. J. Sci. Med. Sport.

[8] Beato M., Drust B., Iacono A.D. (2021). Implementing High-Speed Running and Sprinting Training in Professional Soccer. Int. J. Sports Med..

[9] Duhig S., Shield A.J., Opar D., Gabbett T.J., Ferguson C., Williams M. (2016). Effect of High-Speed Running on Hamstring Strain Injury Risk. Br. J. Sports Med..

[10] Riboli A., Coratella G., Rampichini S., Cé E., Esposito F. (2020). Area per Player in Small-Sided Games to Replicate the External Load and Estimated Physiological Match Demands in Elite Soccer Players. PLoS ONE.

[11] Chaize C., Allen M., Beato M. (2024). Physical Performance Is Affected by Players’ Position, Game Location, and Substitutions During Official Competitions in Professional Championship English Football. J. Strength Cond. Res..

[12] Beato M., Youngs A., Costin A.J. (2024). The Analysis of Physical Performance During Official Competitions in Professional English Football: Do Positions, Game Locations, and Results Influence Players’ Game Demands?. J. Strength Cond. Res..

[13] Castillo-Rodríguez A., González-Téllez J.L., Figueiredo A., Chinchilla-Minguet J.L., Onetti-Onetti W. (2023). Starters and Non-Starters Soccer Players in Competition: Is Physical Performance Increased by the Substitutions?. BMC Sports Sci. Med. Rehabil..

[14] Sarmento H., Martinho D.V., Gouveia É.R., Afonso J., Chmura P., Field A., Savedra N.O., Oliveira R., Praça G., Silva R. (2024). The Influence of Playing Position on Physical, Physiological, and Technical Demands in Adult Male Soccer Matches: A Systematic Scoping Review with Evidence Gap Map. Sports Med..

[15] Hill-Haas S.V., Dawson B., Impellizzeri F.M., Coutts A.J. (2011). Physiology of Small-Sided Games Training in Football: A Systematic Review. Sports Med..

[16] Jäger J., Rumpf M.C., Lochmann M. (2025). The Influence of Relative Pitch Size on External Load in Professional Men’s Soccer. Int. J. Sports Sci. Coach..

[17] Rumpf M., Jäger J., Clemente F., Altmann S., Lochmann M. (2025). The Effect of Relative Pitch Size on Physiological, Physical, Technical and Tactical Variables in Small-Sided Games: A Literature Review and Practical Guide. Front. Sports Act. Living.

[18] Clemente F., Praça G., Aquino R., Castillo D., Raya-González J., Rico-González M., Afonso J., Sarmento H., Silva A., Silva R. (2023). Effects of Pitch Size on Soccer Players’ Physiological, Physical, Technical, and Tactical Responses during Small-Sided Games: A Meta-Analytical Comparison. Biol. Sport.

[19] Riboli A., Olthof S.B.H., Esposito F., Coratella G. (2022). Training Elite Youth Soccer Players: Area per Player in Small-Sided Games to Replicate the Match Demands. Biol. Sport.

[20] Gómez-Carmona C.D., Gamonales J.M., Pino-Ortega J., Ibáñez S.J. (2018). Comparative Analysis of Load Profile between Small-Sided Games and Official Matches in Youth Soccer Players. Sports.

[21] De Dios-Álvarez V., Castellano J., Padrón-Cabo A., Rey E. (2023). Do Small-Sided Games Prepare Players for the Worst-Case Scenarios of Match Play in Elite Young Soccer Players?. Biol. Sport.

[22] Toivonen R.-M., Rantalainen T., Walker S. (2025). The Impact of Varying Small-Sided Games’ Pitch Sizes on Replicating Official Match Physical Demands during Effective Playing Time in Male Professional Soccer Players. Int. J. Perform. Anal. Sport.

[23] Sydney M.G., Wollin M., Chapman D.W., Ball N., Mara J.K. (2022). Do Conditioning Focused Various-Sided Training Games Prepare Elite Youth Male Soccer Players for the Demands of Competition?. Biol. Sport.

[24] Mandorino M., Kavanagh R., Tessitore A., Persichetti V., Morabito M., Lacome M. (2026). “How Many Minutes Does the Player Have in His Legs?” Answering One of Football’s Oldest Coaching Questions Through a Mathematical Model. Appl. Sci..

[25] Mandorino M., Tessitore A., Lacome M. (2024). Loading or Unloading? This Is the Question! A Multi-Season Study in Professional Football Players. Sports.

[26] Winter E.M., Maughan R.J. (2009). Requirements for Ethics Approvals. J. Sports Sci..

[27] Crang Z.L., Duthie G., Cole M.H., Weakley J., Hewitt A., Johnston R.D. (2024). The Validity of Raw Custom-Processed Global Navigation Satellite Systems Data during Straight-Line Sprinting across Multiple Days. J. Sci. Med. Sport.

[28] Mackay L., Sawczuk T., Jones B., Darrall-Jones J., Clark A., Whitehead S. (2025). The Reliability of a Commonly Used (CatapultTM Vector S7) Microtechnology Unit to Detect Movement Characteristics Used in Court-Based Sports. J. Sports Sci..

[29] Cormier P., Tsai M.-C., Meylan C., Agar-Newman D., Epp-Stobbe A., Kalthoff Z., Klimstra M. (2023). Concurrent Validity and Reliability of Different Technologies for Sprint-Derived Horizontal Force-Velocity-Power Profiling. J. Strength Cond. Res..

[30] Cotteret C., González-de-la-Flor Á., Prieto Bermejo J., Almazán Polo J., Jiménez Saiz S.L. (2025). A Narrative Review of the Velocity and Acceleration Profile in Football: The Influence of Playing Position. Sports.

[31] Bates D., Mächler M., Bolker B., Walker S. (2015). Fitting Linear Mixed-Effects Models Using Lme4. J. Stat. Softw..

[32] Winter B. (2013). Linear Models and Linear Mixed Effects Models in R with Linguistic Applications. arXiv.

[33] Cohen J. (1988). Statistical Power Analysis for the Behavioral Sciences.

[34] Martin-Garcia A., Castellano J., Diaz A.G., Cos F., Casamichana D. (2019). Positional Demands for Various-Sided Games with Goalkeepers According to the Most Demanding Passages of Match Play in Football. Biol. Sport.

[35] Beato M., Vicens-Bordas J., Peña J., Costin A.J. (2023). Training Load Comparison between Small, Medium, and Large-Sided Games in Professional Football. Front. Sports Act. Living.

[36] Riboli A., Esposito F., Coratella G. (2023). Technical and Locomotor Demands in Elite Soccer: Manipulating Area per Player during Small-Sided Games to Replicate Official Match Demands. Biol. Sport.

[37] Dello Iacono A., McLaren S.J., Macpherson T.W., Beato M., Weston M., Unnithan V.B., Shushan T. (2023). Quantifying Exposure and Intra-Individual Reliability of High-Speed and Sprint Running During Sided-Games Training in Soccer Players: A Systematic Review and Meta-Analysis. Sports Med..

[38] Chena M., Morcillo-Losa J.A., Rodríguez-Hernández M.L., Asín-Izquierdo I., Pastora-Linares B., Zapardiel J.C. (2022). Workloads of Different Soccer-Specific Drills in Professional Players. J. Hum. Kinet..

[39] Lacome M., Simpson B.M., Cholley Y., Lambert P., Buchheit M. (2018). Small-Sided Games in Elite Soccer: Does One Size Fit All?. Int. J. Sports Physiol. Perform..

[40] Ulupınar S., Özbay S., Ateş O., Turan M., Coşkun K., Çabuk S., Gençoğlu C., Owen A., Barnes C. (2026). An Umbrella Review of Soccer Small-Sided Games: Developing an Evidence-Informed Design Model Linking Training Components to Performance Outcomes. Biol. Sport.

[41] Bortnik L., Nir O., Forbes N., Alexander J., Harper D., Bruce-Low S., Carling C., Rhodes D. (2024). Worst Case Scenarios in Soccer Training and Competition: Analysis of Playing Position, Congested Periods, and Substitutes. Res. Q. Exerc. Sport.

[42] Douchet T., Paizis C., Roche H., Babault N. (2023). Positional Differences in Absolute vs. Relative Training Loads in Elite Academy Soccer Players. J. Sports Sci. Med..

[43] Dalen T., Sandmæl S., Stevens T.G.A., Hjelde G.H., Kjøsnes T.N., Wisløff U. (2021). Differences in Acceleration and High-Intensity Activities Between Small-Sided Games and Peak Periods of Official Matches in Elite Soccer Players. J. Strength Cond. Res..

[44] Moreno-Azze A., Roldán P., Pradas de la Fuente F., Falcón-Miguel D., Gómez-Carmona C.D. (2025). Differences in Accelerations and Decelerations Across Intensities in Professional Soccer Players by Playing Position and Match-Training Day. Appl. Sci..

[45] Owen A.L., Wong D.P., Paul D., Dellal A. (2014). Physical and Technical Comparisons between Various-Sided Games within Professional Soccer. Int. J. Sports Med..

[46] Clemente F., Sarmento H. (2020). The Effects of Small-Sided Soccer Games on Technical Actions and Skills: A Systematic Review. Hum. Mov..

[47] Oliva-Lozano J.M., Gómez-Carmona C.D., Fortes V., Pino-Ortega J. (2021). Effect of Training Day, Match, and Length of the Microcycle on Workload Periodization in Professional Soccer Players: A Full-Season Study. Biol. Sport.

